# Elucidating the multifunctional role of the cell wall components in the maize exploitation

**DOI:** 10.1186/s12870-021-03040-3

**Published:** 2021-06-02

**Authors:** Ana López-Malvar, Rosa Ana Malvar, Xose Carlos Souto, Leonardo Dario Gomez, Rachael Simister, Antonio Encina, Jaime Barros-Rios, Sonia Pereira-Crespo, Rogelio Santiago

**Affiliations:** 1 Facultad, de Biología, Departamento de Biología Vegetal Y Ciencias del Suelo, Universidad de Vigo, As Lagoas Marcosende, 36310 Vigo, Spain; 2 Agrobiología Ambiental, Calidad de Suelos Y Plantas (UVIGO), Unidad Asociada a La MBG (CSIC), Vigo, Spain; 3 Misión Biológica de Galicia (CSIC), Pazo de Salcedo, Carballeira 8, 36143 Pontevedra, Spain; 4 E.E. Forestales, Dpto. Ingenieria Recursos Naturales Y Medio Ambiente, 36005 Pontevedra, Spain; 5 CNAP, Department of Biology, University of York, Heslington, YO10 5DD York UK; 6 Dpto. Ingeniería Y Ciencias Agrarias, Área de Fisiología Vegetal, Universidad de León, Campus de Vegazana s/n, 24071 León, Spain; 7 BioDiscovery Institute and Department of Biological Sciences, University of North Texas, 1155 Union Circle, #311428, Denton, TX 76203-5017 USA; 8 Laboratorio Interprofesional Galego de Análise Do Leite (LIGAL), Mabegondo, 15318 A Coruña, Abegondo Spain

**Keywords:** Maize, Saccharification, Digestibility, Pest resistance, Cell wall

## Abstract

**Background:**

Besides the use of maize grain as food and feed, maize stover can be a profitable by-product for cellulosic ethanol production, whereas the whole plant can be used for silage production. However, yield is reduced by pest damages, stem corn borers being one of the most important yield constraints. Overall, cell wall composition is key in determining the quality of maize biomass, as well as pest resistance. This study aims to evaluate the composition of the four cell wall fractions (cellulose, hemicellulose, lignin and hydroxycinnamates) in diverse maize genotypes and to understand how this composition influences the resistance to pests, ethanol capacity and digestibility.

**Results:**

The following results can be highlighted: (i) pests’ resistant materials may show cell walls with low *p-*coumaric acid and low hemicellulose content; (ii) inbred lines showing cell walls with high cellulose content and high diferulate cross-linking may present higher performance for ethanol production; (iii) and inbreds with enhanced digestibility may have cell walls poor in neutral detergent fibre and diferulates, combined with a lignin polymer composition richer in G subunits.

**Conclusions:**

Results evidence that there is no maize cell wall ideotype among the tested for optimal performance for various uses, and maize plants should be specifically bred for each particular application.

**Supplementary Information:**

The online version contains supplementary material available at 10.1186/s12870-021-03040-3.

## Background

Accessibility, extensibility, and digestibility of tissues would determine important characteristics of maize, such as resistance to stem borers, stem diseases, feedstuff quality and saccharification for ethanol production. These characteristics depend on the cell wall composition and structure [1]. Genetic variation for resistance to corn borers [2], for cell wall composition and degradability [3], and for ethanol production [4] has been identified in maize.

Maize inbred lines have different mechanisms of defence (i.e. morphological plant traits, antibiotic compounds, cell wall stiffening, etc.) that determine the level of corn borer resistance in a particular inbred line [5]. Similarly, saccharification yields or cell wall degradability depend on the cell wall composition and properties of the inbred line, and their response to pre-treatment and hydrolysis [6]. Therefore, it seems that different genotypes may have different defence mechanisms, react differently to pre-treatment, and have different cell wall properties that facilitate saccharification or digestibility.

Maize breeding strategies are normally focused on a particular trait (pest resistance, digestibility or ethanol production) that produce indirect effects on cell wall components. For example, recurrent selection to improve resistance to corn borer attack increases cell wall bound hydroxycinnamates concentration in the pith tissues of the improved maize cycles [7]. Buendgen et al. [8] recorded increases in neutral and acid detergent fibres values (NDF, ADF) and lignin content in the leaf sheath and stalks of maize after five cycles of selection for first and second generation European Corn Borer resistance. Similarly, Bergvinson et al. [9] found increases in diferulic acid concentration over diverse cycles of selection to increase corn borer resistance.

In switchgrass, Sarath et al. [10] studied divergent breeding generations for high or low in vitro dry matter digestibility (IVDMD). This selection resulted in populations differing in lignin concentration and acid detergent fibre. Plants from these two populations also displayed different cell wall composition and accessibility to hydrolytic enzymes [11], which led to an increase in the net mean ethanol yields in plants selected for increased IVDMD. Similarly, after six generations of divergent breeding for forage IVDMD on switchgrass, Vogel et al. [12] observed differences between populations cell wall fibres, lignin content matrix polysaccharides and esterified ferulates. In the same way, selection for improved saccharification efficiency in alfalfa stems produced increases in glucose release and a reduced lignin content in the lines selected [13].

Direct changes towards cell wall structure can also influence the final uses of maize. Successful divergent selection for diferulate ester content in the maize pith resulted in changes in cell wall composition [14]. These changes led to cell wall stiffening, which is the main deterrent of corn borer attack and development. However, the increase in diferulate esters was associated with a decrease in glucose concentration [15]. Furthermore, increasing diferulate content in the maize pith impact negatively in cell wall degradability properties. Jung et al. [16] studied the effect of ferulate crosslinking on dry matter intake, milk production potential and in vivo digestibility for silage maize using the low ferulate seedling ferulate ester (*sfe)* mutant. Lines presenting the mutation showed reduced ether ferulate crosslinking of lignin to arabinoxylans. Cattle fed with this mutant showed greater dry matter intake and milk production.

A final approach to investigate the relationship between cell wall and maize uses consists in mutant or transgenic evaluations. Brown midrib mutants (*bm* 1–4) have been an excellent material to study digestibility and lignification [17]. These mutants have favourable phenotypic traits such as decreased lignin concentration, shift in lignin composition and modified ratio of esterified phenolics. This change in cell wall composition produces higher digestibility and a higher rate of ingestion when fed to cattle [18]. Furthermore, modifications of the phenylpropanoid pathway, through up and downregulation of enzymes directly influencing lignin content and monolignol composition, have been associated with increases in saccharification efficiency in several crops. For example, transgenic switchgrass lines with reduced Cinnamyl Alcohol Dehydrogenase (CAD) levels and consequently reduced lignin content and altered lignin composition, showed improved sugar release and forage digestibility [19].

Considering that limited cell wall components have been tested in specific and limited studies, the aim of the current study is to extensively characterize the cell wall composition and the performance of maize genotypes for different applications using the same experimental trials. The present work has the advantage of including an extensive characterization of the cell wall in a genetically diverse selection of 20 inbred lines. This would help us to confirm previously established relationships in more limited genetic environments. The composition of the cell wall (cellulose, hemicellulose, lignin and hydroxycinnamates) was determined in all inbred lines, and the aptitude for different uses (forage digestibility, ethanol production, digestibility and saccharification) was evaluated.

The main objective of this research is to know the composition of the four cell wall fractions in different maize genotypes and elucidate how this composition influences pest resistance (R), ethanolic production (E) and forage digestibility (D). Those last three aspects will be further on denoted as R.E.D.

## Methods

### Plant material

A set of 20 inbred lines was evaluated (Table 1). These inbreds were divided into groups according to the reason they were included in the panel set:

1. The first subset of inbred lines includes maize inbreed lines that have been characterised in relation to resistance to corn borer attack [2]. Among these inbred lines there are susceptible (EP42, EP47) and resistant (EP17, EP53, EP105, EP125, EP86, F473, PB130, A509) lines. The resistant group (with the exception of EP105) was composed by seven of the eight founders of a Multi-Parent Advanced Generation Inter-Cross (MAGIC) population [20–22]. EP105 derived from the cross A671 x A295 to obtain reduced tunnel length [23]. Lines that are resistant to corn borer attack, show distinct defence mechanisms [24]. This subset of inbred lines was obtained and granted by Mision Biológica de Galicia’s germplasm bank.
2. The second subset includes five inbred lines from the Agriculture and Agri-Food Canada (AAFC): C103, CO384, CO348, CO442 and CO444. Inbred C103 has high concentration of sucrose in the stalk. Reid et al. [25] developed hybrids combinations using C103 as male with CO348, CO384, CO442 and CO444. Authors proposed the usage of these hybrids for ethanol production and biomass for silage, and they are known as “Sugarcorn Hybrids”. This subset of inbred lines was obtained and granted by Dr. Lana Reid from the Eastern Cereal and Oilseed Research Centre in Canada.
3. The last group was formed by inbred lines with good performance in hybrid combination. Inbred EC212 has very good general and specific combinatorial aptitude for high animal digestibility [26]. This group was completed with inbreds A654 and W182B, both belonging to the Reid germplasm group. The hybrid combination attending to heterotic patterns is well stablished in maize; in this case, we would expect good hybrid combinations with the MAGIC inbred parents, these not belonging to Reid germplasm. The inbred EC212 was granted by Dr. Jesus Moreno from the CIAM’s germplasm bank in Mabegondo and A654 and W182 by Misión Biologica de Galicia’s germplasm bank.

**Table 1:** Description (grain type and colour, pedigree, research interest) of the inbred lines included in the experimental set

Inbred line	Germplasm group	Grain colour and type	Pedigree	Research interest
EP17	European	- Yellow - Flint	J.L. Blanco (A1267)	- Parent MAGIC population - Corn borer resistant
EP42	European	- Yellow - Flint	Tomiño. Spanish Landrace	- Corn borer susceptible
EP47	Longfellow	- Yellow - Flint	(EP4 × A239) EP4	- Corn borer susceptible
EP53	European	- Yellow - Flint	Laro. Spanish Landrace	- Parent MAGIC population - Corn borer resistant
EP105	Lancaster	- Yellow - Dent	A671xA295	- Corn borer resistant
EP125	Corn Belt	- Yellow - Dent	Seleccion of CO125. Wisc. exp. single cross	- Parent MAGIC population - Corn borer resistant
EP86	European	- Yellow - Flint	Nostrano dell'Isola. Italian Landrace	- Parent MAGIC population - Corn borer resistant
F473	European	- White - Flint	Doré de Gomer. French Landrace	- Parent MAGIC population - Corn borer resistant
PB130	European	- Yellow - Flint	Rojo vinoso de Aragón. Spanish Landrace	- Parent MAGIC population - Corn borer resistant
A509	Corn Belt	- Yellow - Dent	A78 × A109	- Parent MAGIC population - Corn borer resistant
CO348	Tropical	- Yellow - Flint	CIMMYT-NTR-2	- Sugar corn ethanol hybrids - Moderate *Fusarium* stalk rot resistance
C103	Lancaster	- Yellow - Dent	Lancaster	- High stalk sucrose inbred
CO384	Reid	- Yellow - Flint	A632 x CO255	- Parent of sugar corn ethanol hybrids
CO442	Iodent	- Flint-Dent	Iodent/NSS	- Parent of sugar corn ethanol hybrids
CO444	Lancanster-European	- Yellow - Flint-Dent	S1381 × CO382	- Parent of sugar corn ethanol hybrids - Lodging resistance
EC212	European	- Yellow - Flint	European Germplasm	- High degradability - Good general combining ability (GCA)
A654	Reid	- Yellow - Dent	A116 × WF9	- Good GCA
W182B	Reid	- Yellow - Dent	WD × W22	- Good GCA
A632	Reid	- Yellow - Dent	(Mt42 × B14) B143	- Reid Germplasm Group
W64A	Reid	- Yellow - Dent	WF9 × C.I.187–2	- Corn Borer resistant

### Field experimental design

The inbred panel was evaluated in 2016 and 2017 in Pontevedra, in North Western Spain (42°25′ N, 8°38′ W and 20 m above sea level). A random block design with three repetitions was used in the two trials. The second year, the subset of inbred lines was reduced to nineteen due to the lack of seed for the inbred PB130. Each experimental plot consisted of three rows, each row with 15 double-kernel hills planted manually, spacing between consecutive hills in a row being 0.18 m and 0.8 m between rows, obtaining a final density of ~ 70,000 plants ha^−1^ after thinning. Local agronomical practices were fulfilled.

### Agronomic traits

Por each plot, the days from planting to the date when half of the plants shed pollen or showed silks were recorded as days to anthesis or silking, respectively. Grain and stover yields were recorded at harvest (approx. 70 days after silking); meanwhile forage yield that was recorded 55 days after silking.

#### Grain yield

Grain yield was calculated with the weight of ears collected in a plot and expressed in Mg ha^−1^ at 14% humidity. It was determined by the following equation:

$$Grain\;Yield\left( {\frac{Mg}{{ha}}} \right) = \frac{{Plot\;ear\;weight\;\left( {kg} \right) \times \left( {100 - Humidity} \right)\; \times Grain\;weight\;of\;5\;ears\left( g \right)}}{{Number\;of\;plants\; \times 0.8 \times 0.18\left( {m2} \right) \times 8.6 \times \;Total\;weight\;of\;5\;ears\left( g \right)}}$$

where humidity, expressed as a percentage, was recorded using a moisture meter Kett (model PM-400) in a sample of 240 cm^3^.The value 8.6 on the equation corresponds to a constant to adjust the yield at 14% humidity. Therefore, yield was calculated per plant and then transformed to Mg ha^−1^.

#### Stover and forage yield

In each plot, the weight of 2–10 plants without ears (weight of fresh stover) was recorded, and a stover sample was collected for estimating the percentage of stover dry matter and saccharification efficiency analyses. The fresh stover sample was weighed (sample fresh weight), chopped, pre-dried at 35 °C in a forced air camera, dried at 60 °C in a stove and again weighed after seven days (sample dry weight). Dry stove samples from each plot were grounded in a Wiley mill with a 0.75 mm screen for saccharification assays. Forage yield and forage dry matters were similarly computed but using complete plants collected 55 days after silking.

Stover and forage yields in Mg of dry matter ha^−1^ were determined by the following equation:

$$Stover/ forage\kern0.5em yield\left(\frac{Mg}{ha}\right)=\frac{weigth\kern0.5em of\kern0.5em plants\kern0.5em (g)\ast \kern0.5em sample\kern0.5em dry\kern0.5em weigth(g)}{Number\kern0.5em of\kern0.5em plants\kern0.5em \times 0.8\times 0.18\ast \kern0.5em sample\kern0.5em fresh\kern0.5em weight(g)\times 100}$$

As for grain yield, yields were calculated per plant and then transformed to Mg / ha.

### Cell wall biochemical characterization

The second internode below the main ear was collected from five plants in each plot. Samples were collected 55 days after silking. Each harvested internode was frozen at − 20 °C until analytical determinations. Then, samples were dried at 60 °C and ground in a Wiley (Arthur H. Thomas, Philadelphia, PA) mill with a 0.75 mm screen before being analysed.

Determinations were based in whole dry internode tissues unless mentioned otherwise. Some other biochemical traits (cellulose, total and neutral sugars, uronic acids, and lignin monomeric composition) were determined in isolated cell walls from those internodes following the cell wall isolation protocol modified by Melida et al. [27].

#### Cellulose quantification

Cellulose was quantified by the Updegraff method [28]. The concentration of cellulosic sugars was assessed by the method of the anthrone [29] using glucose as standard.

#### Hemicellulose

For total, and neutral sugars and uronic acids, 5 mg of tissue were weighed and hydrolysed at 212 °C by adding 2 ml of 2 M trifluoroacetic acid (TFA) for 1 h. Total sugar content was determined by the phenol–sulphuric method using glucose as standard [30]. Uronic acid contents were determined by the m-hydroxybiphenyl method [31], with glucuronic acid as a standard. Monosaccharide composition of the matrix polysaccharides was performed in whole dry tissues samples using high performance anion exchange chromatography (HPAEC) (Carbopac PA-10; Dionex, Camberley, Surrey, UK) as described previously by Jones et al. [32].

#### Lignin content and lignin monomeric composition

Total lignin content was determined by Klason Lignin protocol [33]. Monomer composition was determined by thioacidolysis followed by Gas chromatography–mass spectrometry (GC–MS) [34]. The thioacidolysis procedure estimates the proportion of lignin subunits linked by the major β-*O*-4 linkages.

#### Hydroxycinnamates

A recently optimized protocol by Santiago and col. [35] was used for cell wall bound hydroxycinnamates quantification [35]. Phenolic standards ferulic acid (FA) and *p-*coumaric acid (PCA) were purchased from Sigma-Aldrich Quimica SL, Madrid, Spain Sigma. The identities of FA dimers were confirmed by a comparison with 5 − 5 standard or published retention times and UV spectra [36]. The total diferulate content (DFAT) was calculated as the sum of the following three identified and quantified DFA isomers: DFA 8–O–4, DFA 5–5-, and DFA 8–5. The DFA 8–5 concentrations were calculated as the sum of 8 − 5-cyclic (or benzofuran)-DFA and 8–5-noncyclic (or open).

#### Fibres composition

Neutral Detergent Fibre (NDF) and Acid Detergent Fibre (ADF) were determined by Near-Infrared Spectroscopy (NIRs). We included a new variation in the hemicellulose estimation using the hemicellulose content in the fibres, as the difference between ADF and NDF. We will refer to this determination in the text as hemicellulose quantified as percentage (%). The spectral information of the dried and grounded (1 mm) samples was obtained using a Foss NIRSystem 6500 monochromator spectrophotometer (Foss NIRSystem, Silver Spring, Washington, USA), located in an isothermal chamber (24 ± 1° C), provided with a rotation module that performs reflectance measurements in the spectral region between 400 and 2500 nm, at 2 nm intervals. The collection of the spectral data and the chemometric analysis of the data was carried out using the WinISI II v program. 1.5 (Infrasoft International, Port Matilda, PA, USA). In order to detect the presence of extrapolations of the prediction model, the identification of “outliers” samples (spectra not represented within the available NIRS calibration group) was performed using the Mahalanobis (GH). Those samples that presented GH values greater than 3, were selected indicating that the sample did not belong to the calibration group [37]. The samples recognized as outliers were analysed by reference methods in the LIGAL laboratory. Subsequently, the reference values were integrated into the corresponding spectral library of the calibration group, expanding and updating the NIRS prediction equations. The outlier samples were analysed in duplicated by wet determinations using reference methods. NDF and ADF were carried out following the procedures proposed by Van Soest and Robertson [38] for NDF, and by Goering and Van Soest [39] for ADF, adapted to the *Fibre*tec System model 1020 digester (Foss Tecator AB, Höganäs, Sweden).

#### Saccharification efficiency

Saccharification assays were performed as described in Gómez et al. [40]. Samples were pre‐treated with 0.5 M NaOH at 90 °C for 30 min, washed four times with 500 μl sodium acetate buffer and subjected to enzymatic digestion (Celluclast CTec2, 7FPU/g) at 50 °C for 8 h. The amount of released sugars (nmol mg^−1^ material^−1^ h^−1^) was assessed against a glucose standard curve using the 3-methyl-2-benzothiazolinone hydrozone method.

#### Digestibility of the organic matter

Digestibility of the Organic Matter (DOM) of the internode was also determined by Near-infrared spectroscopy (NIRs) at LIGAL. The spectral information of the dried and grounded (1 mm) samples was obtained in the same way reported for fibres estimations. The samples recognized as outliers were analysed by the in vitro digestibility procedure described by Tilley and Terry, [41] modified by Alexander and McGowan [42].

### Statistical analysis

#### Analysis of variance

Individual and combined analyses of variance were made for each trait according to the SAS mixed model procedure (PROC MIXED) of the SAS program (version 9.4) [43], except for DOM and fibres as those estimates were un-replicated. The best linear unbiased estimators (BLUES) for each inbred line were calculated based on the combined data for the 2-year analysis. Inbred lines were considered as the fixed effects, while years, replication within years, and lines × year were considered random effects. The comparison of means among inbred lines was carried out using the Fisher's protected least significant difference (LSD.

#### Correlation analysis

Correlation coefficients between R.E.D traits and yield (grain, stover, and forage yields) were calculated using REML estimates according to a published SAS mixed model procedure [43].

#### Contrast analysis

After the variance analysis, the inbred lines were classified in high and low groups according to saccharification efficiency. For digestibility of organic matter, lines were classified qualitatively. The inbred lines were also classified by its resistance or susceptibility to corn borer attack based on previous evaluations of these materials [2]. Resistance to corn borer has been associated to lower tunnel length of galleries. In this case, the groups were only formed by 5 inbred lines in accordance to the results obtained in previous evaluations [2]. With this new dataset, means comparison were performed in order to determine the existence of significant differences in cell wall composition between high and low groups.

#### Multiple linear regression analysis

In order to understand the relationship between cell wall components and the economically important uses of maize (R.E.D), multiple linear regression models were built for saccharification efficiency and DOM using the cell wall components as probable predictors. The stepwise method following the PROC REG procedure in SAS was used [43]. Only the variables with a significance value less than 0.15 were selected.

## Results

### Analysis of variance and means comparisons

#### Agronomic traits

The subset of inbred lines evaluated showed variation for all the agronomic traits tested (Supplementary Table 1). Inbred lines also differed for grain, forage and stover yields. Year x inbred interaction was only significant for grain yield (data not shown.

#### Cell wall composition

The inbred lines included in this study showed differences in cellulose, lignin, and hydroxycinnamates, but not in hemicellulose. Regarding lignin polymer, we found variation not only for lignin content (Klason Lignin), but also for lignin monomeric composition (Supplementary Table 2-5). Variation for hydroxycinnmamic acids among the inbred lines was except for FA whose *p-*value (0.06) was, however, close to the significance level (Supplementary Table 4).

Inbreds only showed significant differences for one hemicellulose related trait, neutral sugars concentration, but genotype × environment interactions were significant for uronic acids, total sugars and arabinose:xylose ratio. We did not find variation for monosaccharide composition among the 20 genotypes evaluated.

#### R.E.D traits

There was large variability for all traits of economic importance. Within trials involving the final use of maize, inbred lines differed significantly for saccharification efficiency ranging from 85.98 nmol mg^−1^ material^−1^ h^−1^ in the inbred CO384 to 107.72 nmol mg^−1^ material^−1^ h^−1^ in the line F473 (Supplementary Table 6). DOM values varied from 53.4% to 63.8%. There were not significant differences among the inbreds for tunnel length.

### Correlations between R.E.D. and yield traits

Correlations between yield traits and R.E.D were performed in order to assess the covariation among traits. Only significant correlations above 0.50 in absolute values are described, but the complete set of correlations is shown in Table 2. The only strong correlation among those traits was observed between stover and forage yields (*r*_p_ = 0.66).

**Table 2:** Phenotypic correlation for R.E.D and yield traits and correlation values using a particular internode or the whole plant material evaluated in 20 inbred lines during two years

	**SACC**	**DOM**	**Grain yield**	**Forage yield**
SACC				
DOM	0.001			
Grain yield	0.16	0.07		
Forage yield	0.00	0.32	0.34	
Stover yield	-0.01	0.42	0.15	0.66

*SACC* Saccharification efficiency (nmol mg material^−1^ h^−1^, *DOM* Digestibility of the organic matter (%); Grain, Forage and stover yield (Mg/h)

### Contrast and multiple linear regression analysis

To understand the relationship between cell wall components and economically important uses of maize, we performed contrast analysis and multiple linear regression analysis. With the data from the analysis of variance, and the previous data for tunnel length, we classified inbred lines in High, Intermediate and Low for the contrast analysis (Tables 3 and 4). For the multiple linear regression models, we analysed saccharification efficiency and DOM as dependent variables. Model for each variable are shown in Table 5.

**Table 3:** Classification of the inbred lines according to the mean values they presented for R.E.D traits evaluated in two years

Inbred line	Saccharification efficiency	Digestibility of organic matter	Tunnel length
A509	Intermediate	Low	Intermediate
A632	Intermediate	Intermediate	Low
A654	High	Intermediate	Low
C103	Low	High	Intermediate
CO348	Intermediate	High	Low
CO384	Low	High	Intermediate
CO442	High	Intermediate	Intermediate
CO444	Low	Intermediate	High
EC2High2	High	High	Intermediate
EP105	Intermediate	Intermediate	Intermediate
EP125	Low	Low	Low
EP17	High	Intermediate	Intermediate
EP42	High	High	High
EP47	Intermediate	Intermediate	High
EP53	Low	Low	Intermediate
EP86	Intermediate	Low	High
F473	High	Low	Intermediate
PB13I0	Intermediate	High	Low
W182B	Low	Low	High
W64A	Intermediate	Intermediate	Intermediate

**Table 4:** Contrast analysis for the high and low groups of inbred lines classified attending to R.E.D traits. Means for cell wall components with significant differences among groups are included

	**Classification group**		
**CW component**	**High**	**Low**	**LSD**
**Tunnel length (cm)**			
PCA (mg/g)	13.10^a^	12.27^b^	0.777
Hemicellulose (%)	30.2^a^	29.5^b^	0.5
**Saccharification efficiency (nmol mg material**^**−1**^** h**^**−1**^**)**			
DFA 8-O-4 (mg/g)	0.088^a^	0.065^b^	0.015
DFA 8–5-b (mg/g)	0.116^a^	0.089^b^	0.015
DFA 8–5 (mg/g)	0.017^a^	0.014^b^	0.017
DFAT (mg/g)	0.350^a^	0.274^b^	0.041
**Digestibility of the organic matter (%)**			
PCA (mg/g)	10.72^a^	13.94^b^	0.991
DFA 8–5-l (mg/g)	0.042^a^	0.060^b^	0.010
DFA 8–5 (mg/g)	0.125^a^	0.164^b^	0.013
DFAT (mg/g)	0.255^a^	0.320^b^	0.017
G (%)	40.42^a^	39.27^b^	0.852
GLUA (mg/g)	2.46^a^	3.43^b^	0.55

*LSD* Least Square Distance, *PCA p-*coumaric acid, *DFA 8–5-l* Diferulic acid 8–5-Linear, *DFA8O4* Diferulic acid 8-O-4, *DFAT* Total diferulic acids, *DFA 8–5* Diferulic acid 8–5, *G* Subunit, *GLUA* Glucuronic Acid

Means followed by a different letter are significantly different (*P* ≤ 0.05)

**Table 5:** Multiple linear regression stepwise model selection and equation for saccharification efficiency and digestibility of organic matter according to the cell wall composition in the internode of 20 inbred lines evaluated during two years

Stepwise selection			
**Saccharification efficiency (nmol mg material**^**−1**^** h**^**−1**^**)**		***R***^**2**^ **Partial**	***R***^**2**^
Cellulose (CEL mg/g)		0.45	0.45
Diferulic 8–5-b		0.16	0.61
**Model**		SACCHARIFICATION = 53.24 + 14.47*DFA 8–5-b + 0.07*CEL	
**Digestibility of the organic matter (%)**		***R***^**2**^ **Partial**	***R***^**2**^
Neutral Detergent Fibre (NDF)		0.83	0.83
Ferulic Acid (FA)		0.06	0.89
Lignin G Subunit (G)		0.02	0.90
**Model**		DOM=67.78+0.24*FA+0.30*G-0.47*NDF	

*R*^*2*^* partial* Percentage of the variance explained by each trait, *R2* Total percentage of the variance explained by the model

Overall, susceptible inbred lines presented higher concentration of hemicellulose and *p-*coumaric acid than resistant inbred lines. Differences between high and low saccharification efficiency groups (E.) were significant for ferulic acid dimers concentration which were larger in the high saccharification efficiency group. Supporting these results, in the multiple linear regression analysis, we identified cellulose and DFA 8–5-b as significantly involved in the variability for saccharification. Regarding to forage feedstocks (D.), highly degradable lines presented lower concentrations of PCA, DFA 5–5, DFA 8–5-l and DFAT, lower proportion of glucuronic acid, and greater proportions of G subunits. In addition, we obtained in the multiple regression model that DOM was negatively affected by the content in NDF, whereas positively affected by FA and G subunits.

## Discussion

### Studying the variability in agronomic, cell wall and R.E.D traits

#### Agronomic traits

The range obtained for days to silking and anthesis indicates that all inbred lines included in the panel were well adapted to the growing conditions at Pontevedra (42°25′ N, 8°38′ W and 20 m above sea level). It is important to highlight two facts from the yield evaluations: (i) the value obtained for each inbred refers to the maximum-potential yield, not to the yield in a plot; and (ii) these values are for inbred lines, not being the standard material to be tested for yield, as it would be hybrid combinations.

#### Cell wall composition

Inbred lines differed significantly for every main cell wall fraction, with the exception of hemicellulose. Regarding lignin polymer, and in concordance with previous studies, S subunit represented the largest proportion, and H subunits the lowest proportion [44]. Variation for hydroxycinnmamic acids among the inbred lines was also found in accordance being, *p-*coumaric acid was the most abundant hydroxycinnamic acid, which is consistent with previous studies [5, 15].

We did not find variation for matrix monosaccharide composition among the 20 genotypes evaluated that could be consequence of lower variability in secondary than in primary cell walls [45]. However, the lack of differences for monosaccharide composition in the second internode does not exclude the existence of variation among inbreds at more specific stalk tissues (pith and rind) as it has been observed previously by Barros-Rios et al. [46].

#### R.E.D traits

Regarding ethanolic production we found variation for saccharification efficiency. Other researchers have studied the saccharification efficiency of several crops species following the same method and pre-treatment; and variability in saccharification efficiency among different genotypes was also found. Species like *Miscanthus*, switchgrass or sugarcane, are promising candidates for the industrial production of biofuel as they present high biomass yields (15–25 Mg/ha), broad geographic adaptation, superior carbon sequestration and efficient nutrient utilization [47]. However, these species cannot be readily implemented on a wide-commercial scale. In contrast, maize is the most important crop in terms of production, 1300 million tons of dry maize stover are produced worldwide [48]; and can potentially supply vast amounts of lignocellulose in the form of agricultural residues (5 Mg/ha) [47]. In view of the results obtained for saccharification efficiency in contrast to other crops, maize arises as an outstanding model able to contribute significantly to the energy industry’s feedstock both in quality and quantity.

Contrarily to expectations, the parental inbreds of hybrids with high soluble sugar content in the stalk (C103, CO348, CO384, CO444 and CO442) did not show the most promising values for saccharification efficiency, except the inbred line CO442 [25]. Conversely, some other inbred lines could be identified as potential candidates to be base material in breeding programs for increasing the saccharification efficiency: A654, EC212, EP17, EP42 and F473. Four of these inbred lines are included in the European Flint Heterotic Group (EC212, EP17, EP42 and F473) and the last one in the Reid Group (A654), so that it is possible to obtain good heterosis for grain production, while getting high saccharification efficiency.

Regarding DOM, the rank obtained is in agreement with the previous values published for maize in vitro digestibility [49]. Digestibility of the organic matter was higher in maize than in sorghum, with values ranging between 40 and 45%; rice straw, ranging from 23.6% to 36.9%; and barley stems, ranging from 32 to 35% [50, 51].

### Relationship among cell wall components and R.E.D. traits

Resistance to corn borer has been associated to shorter galleries produced by the larvae in the stalks [2]; In view of our results a resistant inbred line in the internode would present cell walls with low p-coumaric acid concentration and low hemicellulose content. Inbred lines showing cell walls with high cellulose content, and high diferulate cross-linking as opposed to cell wall reinforced by lignin may present high saccharification efficiency. By last, inbred lines with enhanced digestibility must have a cell wall poor in neutral detergent fibre and diferulic acid cross-links, combined with a lignin polymer richer in G subunits.

Our results relate hemicellulose with pest resistance. Krakowsky et al. [52] suggested a positive correlation between European corn borer stalk tunnelling and stalk and sheath NDF adjusted for ADF. NDF adjusted represents the relatively digestible hemicellulosic fraction. Additionally, Terra et al. [53] found that hemicellulose is partly digested by the larvae of *Erinnyis ello* feeding on *Euphorbia pulcherrima* leaves, as opposed to other cell wall components. A larger proportion of hemicellulose could favour the attack of the insect, while other structural components involved in tissue strengthening, such as lignin, deter the larvae advance [54].

Regarding DOM, high concentration of fibres (cellulose, hemicellulose and lignin) directly affects digestibility by the limitation of the energy intake by the animals [55]. In agreement with our results, Wolf et al. [55] analysed two maize populations (with high and low concentrations of NDF, ADF, lignin, and silica) and found that the population exhibiting low range of NDF also showed greater digestibility.

A greater concentration of glucuronic acid was also detrimental for DOM. Hemicellulose could be a potential repository of fermentable sugars [56], but unlike cellulose, hemicelluloses are not chemically homogeneous. Maize fibre xylan is one of the complex heteroxylans containing β-(1,4)-linked xylose residues [57]. About 80% of the xylan backbone is highly substituted with monomeric side-chains of arabinose or glucuronic acid linked to O-2 and/or O-3 of xylose residues. The effect of hydrolytic enzymes used in processes such as DOM are influenced by the variation of the primary structure of the arabinoxylans [58]. Based on the works of Van Eylen et al. [59] and Appeldoorn et al. [60] reductions in the frequency of acetic acid, uronic acid and arabinose side groups in glucuronoarabinoxylans would concurrently lead to a reduction in the cell wall recalcitrance. The presence of more substitutions in the arabinoxylan chain, particularly glucuronic acids, could interfere the specific mode of action of hydrolytic enzymes, limiting this way the DOM.

PCA differed in concentration between susceptible and resistant inbred lines; susceptible lines presenting a higher concentration of *p-*coumaric acid. This result appears contradictory with previous results, as high concentration of *p-*coumaric acid has been related to resistance to corn borer attack [5]. On the contrary, higher concentrations of PCA are related with lower DMO. Most *p-*coumarate accretion occurs in tandem with lignification and its accumulation can be considered a relevant indicator of lignin deposition. In grasses, lignins are acylated (primarily syringyl units) at the γ-position by *p-*coumarates [61]. Acylation has a marked influence on the bonding mode of S lignin units, on the spatial organization of lignins and, consequently, on their capacity to interact with polysaccharides. It is known that syringyl type lignin forms a more linear structure, [62] with little or no branching and with a lesser degree of polymerization. This lignin structure protects larger proportion of the polysaccharides in the wall from digestion; thus reducing cell wall digestibility [62]. S lignin has been involved in defence against biotic stresses [63–65]. At least in the materials evaluated S-type lignin, indirectly associated to more PCA acetylation, may favour borer susceptibility. Apparent inconsistencies between studies might be due to the type of tissues analysed; for example, in the current work, stalk (pith and rind) was analysed, while only pith tissues were used in previous studies [15, 24].

In our study, also in relation with lignin structure, lines included in the high digestibility group showed greater proportion of G subunits. Mechin et al. [3] observed a decreased S/G ratio, thus a reduced proportion of subunits S in *bm*3 mutants, characterized by its improved stover digestibility. They also observed that the decrease in the proportion of S subunit was balanced by an increase in proportion of G; which could be, among others, one of the reasons of the increased degradability in *bm3* mutants, agreeing with our results.

Lignocellulosic polysaccharides, mainly cellulose (40%), serve as principal substrate for fermentation of cell wall sugars in ethanol [40]; thereby, cell walls richer in cellulose have more sugars to potentially be fermented. Improving the relative content of cellulose was already one of the main strategies towards the development of advanced lignocellulosic feedstocks [18].

During lignification, the ferulic acid and diferulic esters form cross-links through the etherification of the phenolic hydroxyl group to lignin polymers, [66] forming a polysaccharide-lignin matrix that renders cell wall more recalcitrant to enzymatic hydrolysis [66]. The results obtained in the contrast analysis for DOM are consistent with this statement: the most digestible lines are the ones presenting a cell wall less cross-linked by diferulates. Conversely, our findings indicate that a greater concentration of ester diferulates can be associated with an enhanced saccharification efficiency of the maize stalk. However, as pre-treatments, before the saccharification, are essential to enhance the effectivity of hydrolytic enzymes, the pre-treatment used could be responsible for that unexpected association. Among the several types of pre-treatments that could be used, alkaline pre-treatment is appropriate for corn stover and other monocots due to its particular cell wall composition [67]. The cell walls of graminaceous monocots are known to contain alkali-labile ferulate ester cross-links within the hemicellulose [68], as well as high phenolic hydroxyl contents in their lignins, resulting in increased alkali solubility [69], rendering the cell wall highly susceptible to delignification by alkaline pretreatments [6]. As a consequence, mild alkali pre-treatment of grasses such as maize has shown substantial advantages, as these can be employed for both fractionating biomass and generating a pre-treated biomass that is highly amenable to enzymatic hydrolysis [15, 66]. This may also involve cell wall plasticity, so a greater amount of crosslinking elements in the cell wall may replace the deposition of other structural elements, such as lignin, finally favouring deconstruction [70]. A cell wall where the structural reinforcement is mainly due to a greater crosslinking effect mediated by dimers could represent an improvement in the process of obtaining ethanol, considering that, among the samples pre-treatments, saponification, is usually included and would eliminate these compounds [71]. Li et al. [6] found, in a study of a subset of 26 inbred lines of maize, that the pre-treated cell-wall hydrolysis yields were positively correlated with the ferulate released by alkali pre-treatment, indicating that breaking of ferulate cross-links between cell- wall polymers is an important outcome of pre-treatment. This result reinforces the hypothesis that cell walls where the structural support is based in a higher cross-linkage by diferulates could be more favourable to sugar release, but only after the corresponding alkaline pre-treatment. The role of crosslink is still negative for saccharification if not pre-treated. On the other hand, a larger proportion of ester-link diferulates may indicate a lesser proportion of ether-linked diferulates bound to lignin, and therefore less recalcitrant cell walls. However, ether-linked diferulates cannot be precisely determined under the current wet chemistry procedures.

### Is it possible to identify a particular ideotype useful in R.E.D.?

Our results show that it is very difficult to find a maize ideotype presenting a cell wall composition that fits the three requirements of R.E.D, as one combination of components would favour one use and negatively affects the other. For example, that is the case of the diferulic acid content, which affects cell wall digestibility and saccharification efficiency, or borer resistance and saccharification, both in opposite ways. Therefore, we could aim to obtain a putative genotype from this subset of lines with the best combination of cell wall compositional traits to be used in a particular usage. Despite this, among this subset of inbred lines, we found inbred lines that present high saccharification efficiency and high digestibility of the organic matter: EP42 and EC212; however, we must note that both are susceptible to corn borer attack.

## Conclusions

Genotypic variation has been observed for agronomic, cell wall composition and saccharification efficiency in the set of 20 inbred lines analysed. The germplasm represented by these inbred lines is a potential source of variation that can be exploited in the improvement of biofuel production using maize as a resource.

It is not possible to define a cell wall ideotype that simultaneously fits the requirements for the three uses proposed. We have to settle for identifying the inbred lines with the better combination of cell wall traits for each final use of maize, to be used in future and specific breeding programs. According to these results, we can propose a maize cell wall ideotype to each specific area to be improved: (i) borers resistance in the internode may involve cell walls with low *p-*coumaric acid concentration and low hemicellulose content; (ii) saccharification efficiency must be improved by increasing the presence of cellulose and diferulates; (iii) and, to improve digestibility, cell wall must have poor neutral detergent fiber and diferulate cross-links, combined with a lignin polymer richer in G subunits.

## Supplementary Information

**Additional file 1: Supplementary Table 1**. Means for agronomic traits and yields evaluated in 20 inbred lines during two years. **Supplementary Table 2**. Means of cell wall polysaccharides and matrix monosaccharides in 20 inbred lines evaluated during two years. **Supplementary Table 3**. Means of Klason lignin, lignin monomeric composition in 20 inbred lines evaluated during two years. **Supplementary Table 4**. Means of cell wall-bound hydroxycinnamates in 20 inbred lines evaluated during two years. **Supplementary Table 5**. Values for cell wall fibres in 20 inbred lines evaluated during two years. **Supplementary Table 6**. Means for traits related to R.E.D evaluated in 20 inbred lines during for two years.

## Data Availability

The data sets used and/or analysed during the current study will be available upon request to the corresponding author.

## Abbreviations

AAFC: Agriculture and Agri-Food Canada
ADF: Acid Detergent Fibre
ARA: Arabinose
ARAXYL: Ratio Arabinose/Xylose
AX: Arabinoxylan
BLUEs: Best Linear Unbiased estimators
*bm*: Briwn midrib
CAD: Cinnamyl Alcohol Dehydrogenase
CEL: Celullose
DFA 5–5: Diferulic Acid 5–5
DFA 8–5: Diferulic Acid 8–5
DFA 8–5-b: Diferulic Acid 8–5-Benzofuran
DFA 8–5-l: Diferulic Acid 8–5-Linear
DFA 8-o-4: Diferulic Acid 8-O-4
DFAT: Total Diferulic Acids
DOM: Digestibility of Organic Matter
FA: Ferulic Acid
FUC: Fucose
G: Subunit G
GAL: Galactose
GALA: Galacturonic acid
GCA: Good general combining ability
GLU: Glucose
GLUA: Glucuronic Acid
H: Subunit H
HEMTOT: Percentage of Total Hemicellulose
IVDMD: In vitro Dry Matter Digestibility
IVNDFD: In vitro Neutal Detergent Fibre Digestibility
KL: Klason Lignin
LSD: Least Significance Difference
MAGIC: Multiparent-Advanced Generation Intercross
MAN: Mannose
NIRs: Near-Infrared Spectroscopy
NDF: Neutral Detergent Fibre
NSHEMICEL: Neutral Sugars from Hemicellulose
PCA: *p*-Coumaric Acid
PRIN: Principal Component
R.E.D: Pest Resistance, Ethanol production, forage Digestibility
RHA: Rhammanose
rp: Phenotypic Correlation
S: Subunit S
S:G: S:G ratio
SACC: Saccharification Efficiency
TL: Tunnel Length
TSHEMICEL: Total Sugar from Hemicellulose
UAHEMICEL: Uronic Acids from Hemicellulose
XYL: Xylose
