# Supplementary material for: Elucidating the multifunctional role of the cell wall components in the maize exploitation
Source: BMC Plant Biol. 2021 Jun 2;21:251. doi: 10.1186/s12870-021-03040-3 (PMC8170779; doi:10.1186/s12870-021-03040-3)
Supplement: Supplementary file 1 — Additional file 1: Supplementary Table 1. Means for agronomic traits and yields evaluated in 20 inbred lines during two years. Supplementary Table 2. Means of cell wall polysaccharides and matrix monosaccharides in 20 inbred lines evaluated during two years. Supplementary Table 3. Means of Klason lignin, lignin monomeric composition in 20 inbred lines evaluated during two years. Supplementary Table 4. Means of cell wall-bound hydroxycinnamates in 20 inbred lines evaluated during two years. Supplementary Table 5. Values for cell wall fibres in 20 inbred lines evaluated during two years. Supplementary Table 6. Means for traits related to R.E.D evaluated in 20 inbred lines during for two years. [file 12870_2021_3040_MOESM1_ESM.docx]

Supplementary material

| Supplementary Table 1: Means for agronomic traits and yields evaluated in 20 inbred lines during two years. | | | | | | | | | | |
| --- | --- | --- | --- | --- | --- | --- | --- | --- | --- | --- |
| **Inbred line** | **Anthesis** | | **Silking** | | **Grain Yield (Mg/ha)** | | **Forage Yield (Mg/ha)** | | **Stover Yield (Mg/ha)** | |
| A509 | 62 | ^de^ | 62 | ^de^ | 3.37 | ^defg^ | 8.41 | ^fg^ | 1.84 | ^f^ |
| A632 | 71.8 | ^a^ | 71.8 | ^a^ | 6.26 | ^abcd^ | 14.95 | ^ab^ | 5.95 | ^a^ |
| A654 | 61.8 | ^de^ | 61.8 | ^de^ | 4.95 | ^bcdef^ | 9.27 | ^defg^ | 3.26 | ^abcef^ |
| C103 | 72.7 | ^a^ | 72.7 | ^a^ | 3.23 | ^defg^ | 14.17 | ^abc^ | 5.78 | ^a^ |
| CO348 | 65.5 | ^cd^ | 65.5 | ^cd^ | 2.462 | ^fg^ | 11.97 | ^bcde^ | 3.96 | ^abce^ |
| CO384 | 64.8 | ^cd^ | 64.8 | ^cd^ | 7.49 | ^ab^ | 14.13 | ^abc^ | 5.2 | ^abc^ |
| CO442 | 66 | ^bcd^ | 66 | ^bcd^ | 8.25 | ^a^ | 13.65 | ^abc^ | 5.22 | ^ab^ |
| CO444 | 65.5 | ^cd^ | 65.5 | ^cd^ | 4.39 | ^bcdefg^ | 9.66 | ^defg^ | 3.42 | ^abcef^ |
| EC212 | 64.8 | ^cd^ | 64.8 | ^cd^ | 4.47 | ^bcdefg^ | 10.97 | ^cdef^ | 3.04 | ^bcef^ |
| EP105 | 70.2 | ^ab^ | 70.2 | ^ab^ | 4.31 | ^bcdefg^ | 11.89 | ^bcde^ | 3.6 | ^abcef^ |
| EP125 | 59.7 | ^de^ | 59.7 | ^de^ | 5.23 | ^abcdef^ | 9.59 | ^defg^ | 2.44 | ^ef^ |
| EP17 | 74 | ^a^ | 74 | ^a^ | 6.33 | ^abcd^ | 16.5 | ^a^ | 5.53 | ^ab^ |
| EP42 | 65.8 | ^cd^ | 65.8 | ^cd^ | 2.9 | ^efg^ | 8.42 | ^fg^ | 2.48 | ^cef^ |
| EP47 | 63.5 | ^cde^ | 63.5 | ^cde^ | 4.55 | ^bcdefg^ | 12.37 | ^bcd^ | 5.32 | ^ab^ |
| EP53 | 62.3 | ^de^ | 62.3 | ^de^ | 4.95 | ^bcdef^ | 8.39 | ^fg^ | 2.2 | ^ef^ |
| EP86 | 67.3 | ^bc^ | 67.3 | ^bc^ | 3.33 | ^defg^ | 8.45 | ^fg^ | 1.7 | ^f^ |
| F473 | 66.8 | ^bc^ | 66.8 | ^bc^ | 5.47 | ^abcde^ | 8.9 | ^efg^ | 2.37 | ^ef^ |
| PB130 | 63.6 | ^cde^ | 63.6 | ^cde^ | 1.47 | ^g^ | 7.25 | ^g^ | 2.48 | ^cef^ |
| W182B | 63.3 | ^cde^ | 63.3 | ^cde^ | 4.1 | ^cdefg^ | 8.52 | ^fg^ | 2.37 | ^ef^ |
| W64A | 67.5 | ^bc^ | 67.5 | ^bc^ | 6.78 | ^abc^ | 13.35 | ^abc^ | 3.72 | ^abcef^ |
| **LSD ^a^(P>0.05)** | 4.2 | | 4.1 | | 3.24 | | 3.2 | | 2.73 | |
| a: traits with LSD value are significant at the 0.05 significant level, according to Fisher protected LSD method. A hyphen indicates non-significant differences (P>0.05)  Means followed by a different letter are significantly different (P ≤ 0.05) | | | | | | | | | | |

| Supplementary Table 2: Means of cell wall polysaccharides and matrix monosaccharides in 20 inbred lines evaluated during two years. | | | | | | | | | | | | | | | |
| --- | --- | --- | --- | --- | --- | --- | --- | --- | --- | --- | --- | --- | --- | --- | --- |
| **Inbred**  **Line** | **CEL**  **(**mg/g**)** | **UA**  **HEMICEL**  **(**mg/g**)** | **GALA**  **(**mg/g**)** | **GLUA**  **(**mg/g**)** | **NS**  **HEMICEL**  **(**mg/g**)** | **TS**  **HEMICEL**  **(**mg/g**)** | **FUC**  **(**mg/g**)** | **ARA**  **(**mg/g**)** | **RHA**  **(**mg/g**)** | **GLU**  **(**mg/g**)** | **GAL**  **(**mg/g**)** | **XYL**  **(**µg/mg**)** | **MAN**  **(**mg/g**)** | **ARA:XYL** | **HEM**  **TOT**  **(%)** |
| A509 | 413.79 ^bcd^ | 23.94^a^ | 10.32 ^a^ | 3.29 ^a^ | 233.22 | 257.16 ^a^ | 8.46 ^a^ | 9.83 ^a^ | 20.30 ^a^ | 30.02 ^a^ | 1.02 ^a^ | 25.45 ^a^ | 2.57 ^a^ | 0.47 ^a^ | 13.04 ^a^ |
| A632 | 468.27 ^abcd^ | 23.11 ^a^ | 6.75 ^a^ | 2.42 ^a^ | 217.6 | 240.72 ^a^ | 7.01 ^a^ | 9.09 ^a^ | 25.19 ^a^ | 36.99 ^a^ | 1.73 ^a^ | 21.33 ^a^ | 2.20 ^a^ | 0.64 ^a^ | 12.70 ^a^ |
| A654 | 495.78 ^ab^ | 25.70 ^a^ | 11.16 ^a^ | 1.93 ^a^ | 207.2 | 232.89 ^a^ | 5.19 ^a^ | 9.21 ^a^ | 17.67 ^a^ | 29.65 ^a^ | 1.96 ^a^ | 24.47 ^a^ | 2.29 ^a^ | 0.41 ^a^ | 10.61 ^a^ |
| C103 | 480.31 ^abc^ | 22.53 ^a^ | 6.09 ^a^ | 1.66 ^a^ | 206.47 | 229.01 ^a^ | 4.87 ^a^ | 7.31 ^a^ | 30.1 ^a^ | 45.87 ^a^ | 0.32 ^a^ | 20.39 ^a^ | 1.65 ^a^ | 0.45 ^a^ | 11.71 ^a^ |
| CO348 | 419.07 ^abcd^ | 22.43 ^a^ | 8.56 ^a^ | 1.65 ^a^ | 203.70 | 226.31 ^a^ | 6.1 ^a^ | 7.66 ^a^ | 46.56 ^a^ | 60.94 ^a^ | 1.03 ^a^ | 21.26 ^a^ | 2.07 ^a^ | 0.44 ^a^ | 14.27 ^a^ |
| CO384 | 288.92 ^e^ | 21.01 ^a^ | 10.60 ^a^ | 2.67 ^a^ | 178.49 | 199.77 ^a^ | 7.15 ^a^ | 9.6 ^a^ | 33.78 ^a^ | 47.94 ^a^ | 2.33 ^a^ | 25.35 ^a^ | 3.07 ^a^ | 0.49 ^a^ | 14.12 ^a^ |
| CO442 | 385.06 ^cde^ | 24.64 ^a^ | 12.57 ^a^ | 5.07 ^a^ | 225.85 | 247.67 ^a^ | 11.00 ^a^ | 12.18 ^a^ | 26.46 ^a^ | 40.52 ^a^ | 5.08 ^a^ | 26.77 ^a^ | 5.13 ^a^ | 0.59 ^a^ | 15.47 ^a^ |
| CO444 | 419.92 ^abcd^ | 27.13 ^a^ | 7.53 ^a^ | 2.88 ^a^ | 241.4 | 268.53 ^a^ | 7.07 ^a^ | 7.60 ^a^ | 28.29 ^a^ | 39.81 ^a^ | 1.06 ^a^ | 20.44 ^a^ | 2.79 ^a^ | 0.50 ^a^ | 12.39 ^a^ |
| EC212 | 458.73 ^abcd^ | 24.08 ^a^ | 3.61 ^a^ | 3.90 ^a^ | 207.8 | 231.88 ^a^ | 7.35 ^a^ | 8.31 ^a^ | 34.24 ^a^ | 47.66 ^a^ | 1.62 ^a^ | 18.75 ^a^ | 3.1 ^a^ | 0.89 ^a^ | 13.72 ^a^ |
| EP105 | 437.33 ^abcd^ | 24.51 ^a^ | 13.89 ^a^ | 3.82 ^a^ | 204.85 | 229.36 ^a^ | 9.93 ^a^ | 11.4 ^a^ | 17.65 ^a^ | 30.98 ^a^ | 3.55 ^a^ | 26.57 ^a^ | 3.55 ^a^ | 0.46 ^a^ | 13.53 ^a^ |
| EP125 | 377.18  ^de^ | 27.78 ^a^ | 13.28 ^a^ | 2.65 ^a^ | 191.37 | 219.15 ^a^ | 8.96 ^a^ | 11.12 ^a^ | 26.29 ^a^ | 36.80 ^a^ | 3.26 ^a^ | 27.29 ^a^ | 2.23 ^a^ | 0.44 ^a^ | 13.90 ^a^ |
| EP17 | 449.10 ^abcd^ | 26.87 ^a^ | 8.84 ^a^ | 2.87 ^a^ | 224.61 | 251.49 ^a^ | 7.09 ^a^ | 8.98 ^a^ | 13.70 ^a^ | 27.04 ^a^ | 1.89 ^a^ | 23.68 ^a^ | 3.02 ^a^ | 0.49 ^a^ | 10.75 ^a^ |
| EP42 | 426.46 ^abcd^ | 19.18 ^a^ | 8.34 ^a^ | 2.17 ^a^ | 220.26 | 237.56 ^a^ | 5.69 ^a^ | 7.72 ^a^ | 26.13 ^a^ | 41.85 ^a^ | 0.77 ^a^ | 22.97 ^a^ | 1.77 ^a^ | 0.39 ^a^ | 11.82 ^a^ |
| EP47 | 501.80 ^abcd^ | 24.04 ^a^ | 8.76 ^a^ | 3.20 ^a^ | 216.94 | 240.98 ^a^ | 8.43 ^a^ | 10.67 ^a^ | 29.87 ^a^ | 42.38 ^a^ | 3.26 ^a^ | 24.08 ^a^ | 3.44 ^a^ | 0.6 ^a^ | 14.03 ^a^ |
| EP53 | 372.30 ^de^ | 22.44 ^a^ | 7.65 ^a^ | 1.81 ^a^ | 262.75 | 284.47 ^a^ | 7.14 ^a^ | 5.57 ^a^ | 24.98 ^a^ | 27.05 ^a^ | 0.54 ^a^ | 10.84 ^a^ | 2.34 ^a^ | 0.46 ^a^ | 9.35 ^a^ |
| EP86 | 440.34 ^abcd^ | 22.86 ^a^ | 5.95 ^a^ | 4.42 ^a^ | 188.11 | 210.64 ^a^ | 6.4 ^a^ | 18.11 ^a^ | 29.02 ^a^ | 42.95 ^a^ | 10.63 ^a^ | 21.06 ^a^ | 4.16 ^a^ | 2.22 ^a^ | 11.96 ^a^ |
| F473 | 516.67 ^abcd^ | 24.42 ^a^ | 7.13 ^a^ | 3.34 ^a^ | 215.62 | 240.04 ^a^ | 8.46 ^a^ | 10.01 ^a^ | 20.66 ^a^ | 33.13 ^a^ | 3.15 ^a^ | 22.47 ^a^ | 2.15 ^a^ | 0.44 ^a^ | 13.06 ^a^ |
| PB130 | 436.65 ^abcd^ | 30.01 ^a^ | 8.02 ^a^ | 2.55 ^a^ | 181.85 | 211.14 ^a^ | 7.11 ^a^ | 5.78 ^a^ | 24.79 ^a^ | 39.06 ^a^ | 0.16 ^a^ | 17.48 ^a^ | 2.88 ^a^ | 0.50 ^a^ | 11.34 ^a^ |
| W182B | 294.20 ^abcde^ | 20.19 ^a^ | 8.68 ^a^ | 4.21 ^a^ | 238.08 | 252.85 ^a^ | 5.84 ^a^ | 9.99 ^a^ | 17.42 ^a^ | 30.71 ^a^ | 2.77 ^a^ | 23.18 ^a^ | 4.40 ^a^ | 0.59 ^a^ | 10.95 ^a^ |
| W64A | 462.84 ^abcd^ | 25.92 ^a^ | 9.74 ^a^ | 2.27 ^a^ | 179.46 | 205.33 ^a^ | 4.44 ^a^ | 7.52 ^a^ | 18.91 ^a^ | 31.24 ^a^ | 0 ^a^ | 24.47 ^a^ | 1.90 ^a^ | 0.40 ^a^ | 10.28 ^a^ |
| **LSD ^a^**  **(P<0.05)** | 102.65 | - | - | - | 79.07 | - | - | - | - | - | - | - | - | - | - |
| a: traits with LSD value are significant at the 0.05 significant level, according to Fisher protected LSD method.  A hyphen means there are no significant differences among inbred lines for that trait  Means followed by a differnt letter are significantly different (P ≤ 0.05)  CEL: Celullose; UAHEMICEL: Uronic Acids from hemicellulose; GALA: Galacturonic acid; GLUA: Glucuronic acid; NSHEMICEL: Neutral sugars from hemicellulose; TSHEMICEL: Total sugar from hemicellulose; FUC: Fucose; ARA: Arabinose; RHA: Rhammanose;GLU: glucose; GALl:Galactose XYL: Xylose; MAN: Mannose; ARAXYL: Ratio arabinose/xylose; HEMTOT: Percentage of total hemicellulose | | | | | | | | | | | | | | | |

| Supplementary Table 3: Means of Klason lignin, lignin monomeric composition in 20 inbred lines evaluated during two years. | | | | | | | | | | |
| --- | --- | --- | --- | --- | --- | --- | --- | --- | --- | --- |
| **Inbred Line** | **LK (%)** | | **H (%)** | | **G (%)** | | **S (%)** | | **S:G** | |
| A509 | 27.6 | ^bcdef^ | 2.1 | ^def^ | 38.5 | ^hij^ | 59.5 | ^ab^ | 1.6 | ^a^ |
| A632 | 33.6 | ^ab^ | 2.4 | ^de^ | 43.4 | ^ab^ | 54.2 | ^hi^ | 1.3 | ^cd^ |
| A654 | 28.5 | ^bcdef^ | 1.5 | ^f^ | 40.4 | ^defgh^ | 58.1 | ^bcdef^ | 1.4 | ^bc^ |
| C103 | 20.8 | ^ef^ | 2.8 | ^cd^ | 39.7 | ^efgh^ | 57.6 | ^bcdefg^ | 1.5 | ^ab^ |
| CO348 | 19.3 | ^f^ | 1.8 | ^ef^ | 39.4 | ^efghi^ | 58.8 | ^abcd^ | 1.5 | ^ab^ |
| CO384 | 28.2 | ^bcdef^ | 3.7 | ^ab^ | 42.6 | ^bc^ | 53.7 | ^i^ | 1.3 | ^cd^ |
| CO442 | 27.6 | ^bcdef^ | 2 | ^def^ | 40.5 | ^defg^ | 57.5 | ^bcdefg^ | 1.4 | ^bc^ |
| CO444 | 28 | ^bcdef^ | 2.4 | ^de^ | 41.1 | ^cde^ | 56.5 | ^efg^ | 1.4 | ^bc^ |
| EC212 | 26.9 | ^bcdef^ | 1.9 | ^ef^ | 41 | ^cde^ | 57.1 | ^defg^ | 1.4 | ^bc^ |
| EP105 | 32.1 | ^ab^ | 2.2 | ^def^ | 38.8 | ^ghij^ | 59.1 | ^abcd^ | 1.5 | ^ab^ |
| EP125 | 26.7 | ^bcdef^ | 4.3 | ^a^ | 40.9 | ^cdef^ | 54.8 | ^h^ | 1.3 | ^cd^ |
| EP17 | 31.8 | ^abcd^ | 2.5 | ^cde^ | 45.1 | ^ab^ | 52.4 | ^i^ | 1.2 | ^d^ |
| EP42 | 21.8 | ^cdef^ | 1.8 | ^ef^ | 39 | ^fghij^ | 59.2 | ^abc^ | 1.5 | ^ab^ |
| EP47 | 23.1 | ^bcdef^ | 2 | ^def^ | 40.7 | ^cdefg^ | 57.3 | ^cdefg^ | 1.4 | ^bc^ |
| EP53 | 42.2 | ^a^ | 2.5 | ^cde^ | 38.9 | ^ghij^ | 58.6 | ^abcd^ | 1.5 | ^ab^ |
| EP86 | 31.1 | ^bcde^ | 2.6 | ^cde^ | 41.7 | ^bcd^ | 55.8 | ^gh^ | 1.3 | ^cd^ |
| F473 | 21.1 | ^def^ | 3.3 | ^bc^ | 38.4 | ^ij^ | 58.3 | ^abcde^ | 1.5 | ^ab^ |
| PB130 | 19 | ^f^ | 2.3 | ^def^ | 40.5 | ^defg^ | 57.1 | ^defg^ | 1.4 | ^bc^ |
| W182B | 27.9 | ^bcdef^ | 2.6 | ^cde^ | 37.2 | ^j^ | 60.2 | ^a^ | 1.6 | ^a^ |
| W64A | 32.7 | ^ab^ | 1.9 | ^ef^ | 41.9 | ^bcd^ | 56.2 | ^fgh^ | 1.3 | cd |
| **LSD ^a (^P< 0.05)** | 10.7 | | 0.8 | | 1.9 | | 2 | | 0.1 | |
| a: traits with LSD value are significant at the 0.05 significant level, according to Fisher protected LSD method.  A hyphen means there are no significant differences among inbred lines for that trait  Means followed by a differnt letter are significantly different (P ≤ 0.05)  LK: Klason lignin; H: Subunit H; G: Subunit G, S:Subunit S ; S:G: S:G ratio | | | | | | | | | | |

| Supplementary Table 4: Means of cell wall-bound hydroxycinnamates in 20 inbred lines evaluated during two years. | | | | | | | | | | | | | | | | |
| --- | --- | --- | --- | --- | --- | --- | --- | --- | --- | --- | --- | --- | --- | --- | --- | --- |
| **Inbred Line** | **PCA**  **(mg/g)** | | **FA**  **(mg/g)** | | **DFA 5-5**  **(mg/g)** | | **DFA 8-O-4 (mg/g)** | | **DFA8-5-l**  **(mg/g)** | | **DFA 8-5-b**  **(mg/g)** | | **DFA 8-5**  **(mg/g)** | | **DFAT (mg/g)** | |
| A509 | 14.27 | ^abc^ | 3.222 | ^a^ | 0.078 | ^cde^ | 0.059 | ^def^ | 0.067 | ^ab^ | 0.088 | ^efgh^ | 0.154 | ^defgh^ | 0.291 | ^cde^ |
| A632 | 12.17 | ^fghi^ | 3.285 | ^a^ | 0.082 | ^bcde^ | 0.111 | ^a^ | 0.061 | ^abcde^ | 0.083 | ^fgh^ | 0.145 | ^defghi^ | 0.338 | ^bc^ |
| A654 | 15.23 | ^a^ | 3.012 | ^a^ | 0.098 | ^abcd^ | 0.076 | ^bcde^ | 0.053 | ^bcdefg^ | 0.109 | ^def^ | 0.162 | ^defg^ | 0.337 | ^bc^ |
| C103 | 9.272 | ^k^ | 2.665 | ^a^ | 0.061 | ^ef^ | 0.061 | ^def^ | 0.041 | ^efg^ | 0.082 | ^fgh^ | 0.123 | ^ghij^ | 0.244 | ^def^ |
| CO348 | 10.1 | ^jk^ | 3.844 | ^a^ | 0.063 | ^ef^ | 0.07 | ^cdef^ | 0.043 | ^cdefg^ | 0.088 | ^efgh^ | 0.13 | ^fghij^ | 0.262 | ^cdef^ |
| CO384 | 10.8 | ^ij^ | 2.357 | ^a^ | 0.051 | ^f^ | 0.042 | ^f^ | 0.041 | ^efg^ | 0.063 | ^h^ | 0.104 | ^j^ | 0.197 | ^f^ |
| CO442 | 12.94 | ^cdefg^ | 3.599 | ^a^ | 0.105 | ^ab^ | 0.103 | ^ab^ | 0.077 | ^a^ | 0.142 | ^bc^ | 0.219 | ^ab^ | 0.427 | ^a^ |
| CO444 | 12.68 | ^defg^ | 3.068 | ^a^ | 0.079 | ^bcde^ | 0.074 | ^bcdef^ | 0.062 | ^abcd^ | 0.107 | ^def^ | 0.168 | ^cdef^ | 0.321 | ^bcd^ |
| EC212 | 13.63 | ^bcde^ | 3.115 | ^a^ | 0.073 | ^def^ | 0.084 | ^abcd^ | 0.033 | ^g^ | 0.088 | ^efgh^ | 0.121 | ^hij^ | 0.278 | ^cdef^ |
| EP105 | 14.47 | ^ab^ | 3.418 | ^a^ | 0.113 | ^a^ | 0.109 | ^a^ | 0.076 | ^a^ | 0.127 | ^cd^ | 0.203 | ^abc^ | 0.425 | ^a^ |
| EP125 | 13.33 | ^bcdef^ | 2.897 | ^a^ | 0.087 | ^abcde^ | 0.084 | ^abc^ | 0.06 | ^abcde^ | 0.097 | ^efg^ | 0.158 | ^defgh^ | 0.329 | ^bc^ |
| EP17 | 11.68 | ^ghi^ | 3.134 | ^a^ | 0.081 | ^bcde^ | 0.084 | ^abcd^ | 0.063 | ^abc^ | 0.104 | ^def^ | 0.167 | ^cdef^ | 0.333 | ^bc^ |
| EP42 | 11.25 | ^hij^ | 2.628 | ^a^ | 0.07 | ^ef^ | 0.07 | ^cdef^ | 0.039 | ^fg^ | 0.097 | ^efg^ | 0.136 | ^efghij^ | 0.275 | ^cdef^ |
| EP47 | 12.38 | ^efgh^ | 3.754 | ^a^ | 0.102 | ^abc^ | 0.101 | ^abc^ | 0.069 | ^ab^ | 0.116 | ^cde^ | 0.181 | ^bcd^ | 0.384 | ^ab^ |
| EP53 | 13.06 | ^cdef^ | 2.57 | ^a^ | 0.071 | ^ef^ | 0.045 | ^ef^ | 0.038 | ^g^ | 0.068 | ^gh^ | 0.108 | ^ij^ | 0.227 | ^ef^ |
| EP86 | 14.82 | ^ab^ | 2.758 | ^a^ | 0.078 | ^cde^ | 0.053 | ^def^ | 0.044 | ^cdefg^ | 0.081 | ^fgh^ | 0.125 | ^ghij^ | 0.256 | ^cdef^ |
| F473 | 13.84 | ^bcd^ | 3.805 | ^a^ | 0.103 | ^abc^ | 0.114 | ^a^ | 0.07 | ^a^ | 0.157 | ^b^ | 0.226 | ^a^ | 0.442 | ^a^ |
| PB130 | 8.454 | ^k^ | 2.131 | ^a^ | 0.079 | ^bcde^ | 0.075 | ^bcde^ | 0.042 | ^defg^ | 0.951 | ^a^ | 0.139 | ^efghij^ | 0.296 | ^cde^ |
| W182B | 14.15 | ^bc^ | 2.57 | ^a^ | 0.082 | ^bcde^ | 0.071 | ^bcdef^ | 0.061 | ^abcde^ | 0.113 | ^cde^ | 0.174 | ^cde^ | 0.327 | ^bc^ |
| W64A | 13.74 | ^bcde^ | 2.758 | ^a^ | 0.1 | ^abcd^ | 0.068 | ^def^ | 0.059 | ^abcdef^ | 0.1 | ^def^ | 0.158 | ^defgh^ | 0.326 | ^bcd^ |
| **LSD ^a (P< 0.05)^** | 1.374 | | - | | 0.026 | | 0.032 | | 0.02 | | 0.029 | | 0.04 | | 0.084 | |
| a: traits with LSD value are significant at the 0.05 significant level, according to Fisher protected LSD method.  A hyphen means there are no significant differences among inbred lines for that trait  Means followed by a different letter are significantly different (P ≤ 0.05)  PCA: *p-*coumaric acid; FA: Ferulic acid; DFA55: Diferulic acid 5-5; DFA8o4: Diferulic acid 8-O-4, DFA85l: Diferulic acid 8-5-Linear;DFA85b: Diferulic acid 8-5-Benzofuran;DFA85: Diferulic acid 8-5;DFAT: Total diferulic acids. | | | | | | | | | | | | | | | | |

| Supplementary Table 5: Values for cell wall fibres in 20 inbred lines evaluated during two years. | | | |
| --- | --- | --- | --- |
| **Inbred Line** | **ADF**  **(%)** | **NDF**  **(%)** | **Hemicellulose**  **(%)** |
| A509 | 45.8 | 71.7 | 25.9 |
| A632 | 39.2 | 62.2 | 23.0 |
| A654 | 39.5 | 60.3 | 20.8 |
| C103 | 33.0 | 44.2 | 11.2 |
| CO348 | 29.8 | 38.9 | 9.1 |
| CO384 | 30.6 | 46.4 | 15.8 |
| CO442 | 38.4 | 64.0 | 25.6 |
| CO444 | 37.4 | 59.9 | 22.5 |
| EC212 | 34.0 | 53.0 | 19.0 |
| EP105 | 42.3 | 68.8 | 26.5 |
| EP125 | 42.5 | 67.8 | 25.4 |
| EP17 | 41.8 | 65.3 | 23.4 |
| EP42 | 34.2 | 52.0 | 17.8 |
| EP47 | 36.1 | 57.1 | 21.1 |
| EP53 | 39.2 | 58.9 | 19.6 |
| EP86 | 42.3 | 63.3 | 21.0 |
| F473 | 43.6 | 69.5 | 25.9 |
| PB130 | 25.9 | 42.9 | 17.1 |
| W182B | 37.7 | 60.5 | 22.9 |
| W64A | 33.3 | 55.9 | 22.5 |
| ADF: Acid Detergent Fibre; NDF: Neutral Detergent Fibre. | | | |

| Supplementary Table 6: Means for traits related to R.E.D evaluated in 20 inbred lines during for two years. | | |
| --- | --- | --- |
| **Inbred line** | **Saccharification**  **efficiency**  **(nmol mg^-1^ material^-1^ hour^-1^)** | **Digestibility of**  **organic matter**  **(%)** |
| A509 | 96.56 ^bcdef^ | 53.4 |
| A632 | 96.92 ^bcdef^ | 57.6 |
| A654 | 108.96 ^a^ | 59.2 |
| C103 | 93.05 ^ef^ | 67.6 |
| CO348 | 94.72 ^def^ | 68.4 |
| CO384 | 85.98 ^f^ | 64.6 |
| CO442 | 105.05 ^abcd^ | 59.1 |
| CO444 | 93.67 ^def^ | 59.3 |
| EC212 | 102.70 ^abcde^ | 63.8 |
| EP105 | 101.42 ^abcde^ | 57.8 |
| EP125 | 92.95 ^ef^ | 54.5 |
| EP17 | 101.58 ^abcde^ | 59.0 |
| EP42 | 108.25 ^ab^ | 63.0 |
| EP47 | 98.25 ^abcde^ | 62.9 |
| EP53 | 80.18 ^f^ | 56.1 |
| EP86 | 95.68 ^def^ | 55.1 |
| F473 | 107.72 ^abc^ | 55.1 |
| PB130 | 96.28 ^cdef^ | 64.6 |
| W182B | 84.29 ^f^ | 55.2 |
| W64A | 97.87 ^abcde^ | 60.5 |
| **LSD ^a^**  **(P < 0.05)** | 11.81 |  |
| a: traits with LSD value are significant at the 0.05 significant level, according to Fisher protected LSD method.  A blank space in the LSD for DMO indicates that this trait was not included in the variance analysis.  Means followed by a different letter are significantly different (P ≤ 0.05) | | |
